# Validation of rapid shallow breathing index displayed by the ventilator compared to the standard technique in patients with readiness for weaning

**DOI:** 10.1186/s12890-021-01680-7

**Published:** 2021-10-02

**Authors:** Nuttapol Rittayamai, Natwipha Ratchaneewong, Pirat Tanomsina, Withoon Kongla

**Affiliations:** 1 Division of Respiratory Diseases and Tuberculosis, Department of Medicine, Faculty of Medicine Siriraj Hospital, Mahidol University, 2 Wanglang Road, Bangkoknoi, Bangkok, 10700 Thailand; 2 Department of Medicine, Faculty of Medicine Siriraj Hospital, Mahidol University, Bangkok, Thailand; 3 Department of Medicine, Chulabhorn Hospital, HRH Princess Chulabhorn College of Medical Science, Chulabhorn Royal Academy, Bangkok, Thailand

**Keywords:** Mechanically ventilated patients, Rapid shallow breathing index, Ventilator, Wright spirometer, Weaning

## Abstract

**Background:**

Rapid shallow breathing index (RSBI) is the most commonly used parameter for predicting weaning outcome. Measurement of RSBI by Wright spirometer (RSBI_standard_) is the standard method in routine clinical practice. Data specific to the accuracy and reliability of the RSBI value displayed by the ventilator (RSBI_vent_) are scarce. Accordingly, this study aimed to evaluate the association between the average value of RSBI_vent_ at different time points and RSBI_standard_, and to assess the accuracy and reliability of these two RSBI measurement techniques.

**Methods:**

This prospective cohort study included mechanically ventilated patients who were ready to wean. At the beginning of spontaneous breathing trial using the flow-by method, RSBI was measured by two different techniques at the same time, including: (1) Wright spirometer (breathing frequency/average tidal volume in 1 min) (RSBI_standard_), and (2) the values displayed on the ventilator at 0, 15, 30, 45, and 60 s (RSBI_vent_).

**Results:**

Forty-seven patients were enrolled. The RSBI_vent_ value was significantly higher than the RSBI_standard_ value for every comparison. According to Spearman’s correlation coefficient (*r*) and intraclass correlation coefficient (ICC), the average value of RSBI from 5 time points (0, 15, 30, 45, and 60 s) showed the best correlation with the standard technique (*r* = 0.76 [*P* < 0.001], and ICC = 0.79 [95% CI 0.61–0.88], respectively). Bland–Altman plot also showed the best agreement between RSBI_standard_ and the RSBI_vent_ value averaged among 5 time points (mean difference − 17.1 breaths/min/L).

**Conclusions:**

We found that the ventilator significantly overestimates the RSBI value compared to the standard technique by Wright spirometer. The average RSBI_vent_ value among 5 time points (0, 15, 30, 45, and 60 s) was found to best correlate with RSBI_standard_.

## Background

Mechanical ventilation is a life-saving treatment in critically ill patients with acute respiratory failure; however, prolonged mechanical ventilation significantly increases healthcare utilization and cost, and is associated with poor outcomes [1, 2]. Many studies reported that delayed discontinuation of mechanical ventilation increased the risk of ventilator-induced lung injury, ventilator-associated pneumonia, diaphragm myotrauma, and other complications [3–6]. Moreover, patients with prolonged mechanical ventilation have increased intensive care unit (ICU) length of stay and hospital mortality [7, 8].

Liberation or weaning from mechanical ventilation is an important process that can account for 40–60% of the total duration of mechanical ventilation [9, 10], and determination of the appropriate time to initiate weaning can be a challenge. Assessment of readiness to wean is an important step before performing a spontaneous breathing trial (SBT), and patient readiness should be evaluated as soon as possible after the patient recovers from acute respiratory failure. Several parameters have been proposed for predicting the weaning outcome. Rapid shallow breathing index (RSBI), defined as the ratio of breathing frequency to average tidal volume in 1 min (breaths/min/L), has been shown to be one of the most accurate predictors of weaning outcome [11]. A cut-off value greater than 105 breaths/min/L has been used to predict weaning trial failure [12]. RSBI measurement using a Wright spirometer is a gold standard method; however, it requires a special instrument that might not be available at the bedside.

Many modern ICU ventilators measure and display the RSBI value; however, the value displayed on the ventilator may vary breath by breath according to the algorithm used for calculating the RSBI. In addition, ventilator settings, such as pressure augmentation, positive-end expiratory pressure (PEEP), and a bias flow may also affect ventilator measurement of the RSBI. Data specific to the accuracy and reliability of the RSBI value displayed by the ventilator (RSBI_vent_) compared with standard measurement of RSBI using a Wright spirometer (RSBI_standard_) are limited. Accordingly, the aim of this study was to evaluate the correlation between the average value of RSBI_vent_ at different time points and RSBI_standard_, and to assess the accuracy and reliability of these two RSBI measurement techniques. We hypothesized that using the average value of RSBI_vent_ among different time points would increase the evaluated performance parameters compared to RSBI_standard_.

## Methods

### Study design and population

This prospective cohort study included mechanically ventilated patients who were admitted to the Division of Respiratory Diseases and Tuberculosis of the Department of Medicine, Faculty of Medicine Siriraj Hospital, Mahidol University, Bangkok, Thailand during June 1st, 2019 to December 15th, 2019. The study protocol was approved by the Siriraj Institutional Review Board (SIRB) (certificate of approval no. 275/2018), and was registered in the Thai Clinical Trial Registry (#TCTR20180606001). Written informed consent was obtained from each subject or their relatives.

Patients meeting all of the following criteria were eligible for inclusion: (1) age from 18–90 years; (2) on mechanical ventilation > 24 h; and, (3) readiness to wean with all of the following[13]: stable hemodynamics (heart rate < 140 beats/min, systolic blood pressure within 90–160 mmHg, receiving no or low-dose vasopressor [equivalent to dopamine < 5 mcg/kg/min]), adequate oxygenation (oxygen saturation by pulse oximetry [SpO_2_] ≥ 92% or arterial partial pressure of oxygen (PaO_2_)/inspired oxygen fraction (FiO_2_) ≥ 200 mmHg) with pressure support ≤ 12 cmH_2_O, FiO_2_ ≤ 0.5, and PEEP ≤ 8 cmH_2_O, and adequate mental status (Glasgow Coma Scale ≥ 13 and not receiving sedative drugs). Patients with tracheostomy tube, pregnant women, and uncooperative patients were excluded.

### Study protocol

All subjects were ventilated using a VELA ventilator (Vyaire Medical, Inc., Mettawa, IL, USA) with head-of-bed elevation of 30°–45° throughout the study. Ventilators and circuits were calibrated to prevent measurement bias. Subjects were stabilized and continuously monitored for blood pressure, heart rate, breathing frequency, and SpO_2_ during the study period. Endotracheal aspiration of secretion was performed before starting the study protocol.

**Fig. 1:**
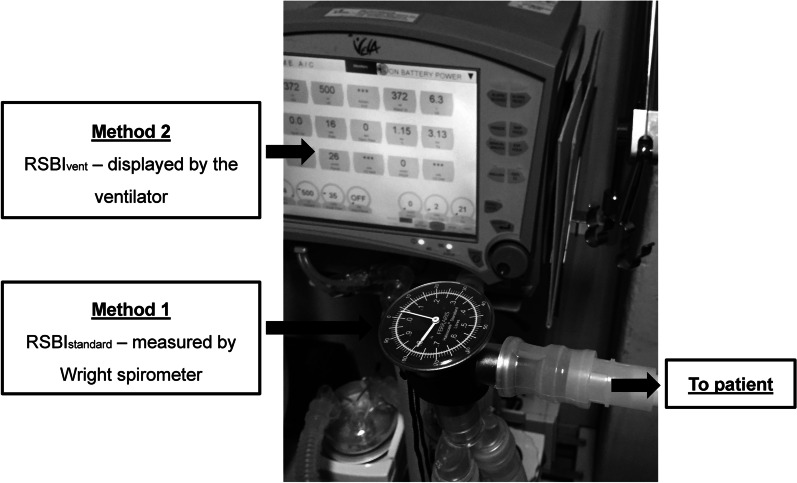
Measurement of rapid shallow breathing index (RSBI) by Wright spirometer (Method 1; RSBI_standard_), and RSBI measured and displayed by the ventilator (Method 2; RSBI_vent_)

RSBI was measured at the beginning of SBT with a flow-by technique using the following ventilator settings: pressure support of 0 cmH_2_O, PEEP of 0 cmH_2_O, FiO_2_ of 0.4, flow trigger of 2 L/min, and bias flow of 10 L/min. The “Ferraris Haloscale” Wright spirometer (Ferraris Medical, Inc., Louisville, CO, USA) was attached between endotracheal tube and Y-piece. Respiratory variables measured by the ventilator (i.e. tidal volume, breathing frequency, minute ventilation, and RSBI) were selected to show on the ventilator screen (Fig. 1). After the patient began spontaneous breathing with a flow-by technique for one minute, the two RSBI measurement techniques including RSBI_standard_ and RSBI_vent_ were simultaneously measured for one minute by the same examiner (NAR). RSBI_standard_ was defined as the standard method using Wright spirometer. RSBI_vent_ was defined as the RSBI value measured and displayed by the ventilator. RSBI measurement was terminated if the subject met at least one of the following criteria: SpO_2_ < 92%, breathing frequency ≥ 35 breaths/minute, heart rate ≥ 140 beats/min, malignant arrhythmia, or systolic blood pressure < 90 or > 160 mmHg. After completing the study protocol, the ventilator was returned to its pre-test settings.

### Data collection

Baseline characteristics that were recorded included age, gender, Acute Physiologic and Chronic Health Evaluation (APACHE) II score, comorbidity, type of respiratory failure, the principal diagnosis, and duration of mechanical ventilation. RSBI_standard_ measured by Wright spirometer was calculated as the breathing frequency divided by average tidal volume (in liters) in 1 min. The RSBI_vent_ value was recorded from the ventilator screen at 0, 15, 30, 45, and 60 s at the same time of measuring RSBI_standard_ using Wright spirometer in 1 min. The average value of RSBI at different time points, including 0–15, 0–30, 0–45, and 0–60 s, was calculated.

### Outcomes

The study outcomes were (1) the correlation between the average value of RSBI_vent_ at different time points and RSBI_standard_, and (2) the accuracy and reliability of RSBI measurement by the two techniques.

### Statistical analysis

Using data from a previous study [14], a 2-sided α value of 0.05, and a power of 80% a sample size of 47 patients was calculated to evaluate the accuracy and reliability between the two RSBI measurement techniques. Normality of data distribution was tested by Kolmogorov–Smirnov test. Continuous data are presented as mean ± standard deviation or median [interquartile range] as appropriate. Categorical variables are presented as absolute number and percentage. We performed a correlation analysis between the two techniques using Spearman’s correlation coefficient (*r*) and intraclass correlation coefficient (ICC). Bland–Altman plot was used to evaluate the limit of agreement between RSBI_vent_ and gold standard RSBI_standard_. A *P* value less than 0.05 was considered statistically significant. Statistical analysis was performed using PASW Statistics 18 (SPSS, Inc., Chicago, Illinois, USA) and MedCalc Statistical Software (Ostend, Belgium).

## Results

Forty-seven mechanically ventilated subjects were enrolled. The median [interquartile range] age of subjects was 68.0 [60.0–75.0] years, 57.4% of them were female, and APACHE II score was 18.0 [17.0–20.0]. Pneumonia was the leading cause of hospital admission (44.7%) followed by congestive heart failure (19.1%) and extrapulmonary sepsis (17.0%). Other baseline characteristics are presented in Table 1.

**Table 1:** Baseline demographics and clinical characteristics

Variables	N = 47
Age, years	68.0 [60.0–75.0]
Female, n (%)	27 (57.4%)
Body mass index, kg/m^2^	22.0 [20.8–24.0]
Comorbidity, n (%)	
Hypertension	31 (66.0%)
Diabetes mellitus	19 (40.4%)
Cardiovascular disease	10 (21.3%)
Respiratory disease	7 (14.9%)
Chronic kidney disease	9 (19.1%)
Chronic liver disease	1 (2.1%)
Malignancy	8 (17.0%)
Others	6 (12.8%)
APACHE II at enrollment	18.0 [17.0–20.0]
Principal diagnosis on admission, n (%)	
Pneumonia	21 (44.7%)
Congestive heart failure	9 (19.1%)
Extrapulmonary sepsis	8 (17.0%)
Acute exacerbation of COPD	5 (10.6%)
Others	4 (8.3%)
Type of acute respiratory failure, n (%)	
Hypoxemic	34 (72.4%)
Hypercapnic	5 (10.6%)
Sepsis	8 (17.0%)

Data are presented as median [interquartile range] or n (%)

*APACHE II* Acute Physiology and Chronic Health Evaluation II, *COPD* chronic obstructive pulmonary disease

The median [interquartile range] RSBI_standard_ value was 66.0 [38.2–90.0] breaths/min/L. The average RSBI_vent_ values at different time points are shown in Table 2. For all comparisons, the RSBI_vent_ value was significantly higher than the RSBI_standard_ value (Table 2).

**Table 2:** Rapid shallow breathing index value measured by Wright spirometer and the average rapid shallow breathing index value displayed by the ventilator at different time points

RSBI_standard_, breaths/min/L	Median [interquartile range]	Average RSBI_vent_ at different time points, breaths/min/L	Median [interquartile range]	*P* value
	66.0 [38.2–90.0]	At 0–15	85.5 [58.5–118.5]	< 0.001
	66.0 [38.2–90.0]	At 0–30	81.7 [61.0–114.7]	< 0.001
	66.0 [38.2–90.0]	At 0–45	79.5 [55.3–112.3]	< 0.001
	66.0 [38.2–90.0]	At 0–60	79.2 [57.4–113.0]	< 0.001

Data are presented as median [interquartile range]

*RSBI*_*standard*_ rapid shallow breathing index measured by Wright spirometer, *RSBI*_*vent*_ rapid shallow breathing index displayed by the ventilator

### Correlation and limit of agreement between RSBI_standard_ and RSBI_vent_

**Fig. 2:**
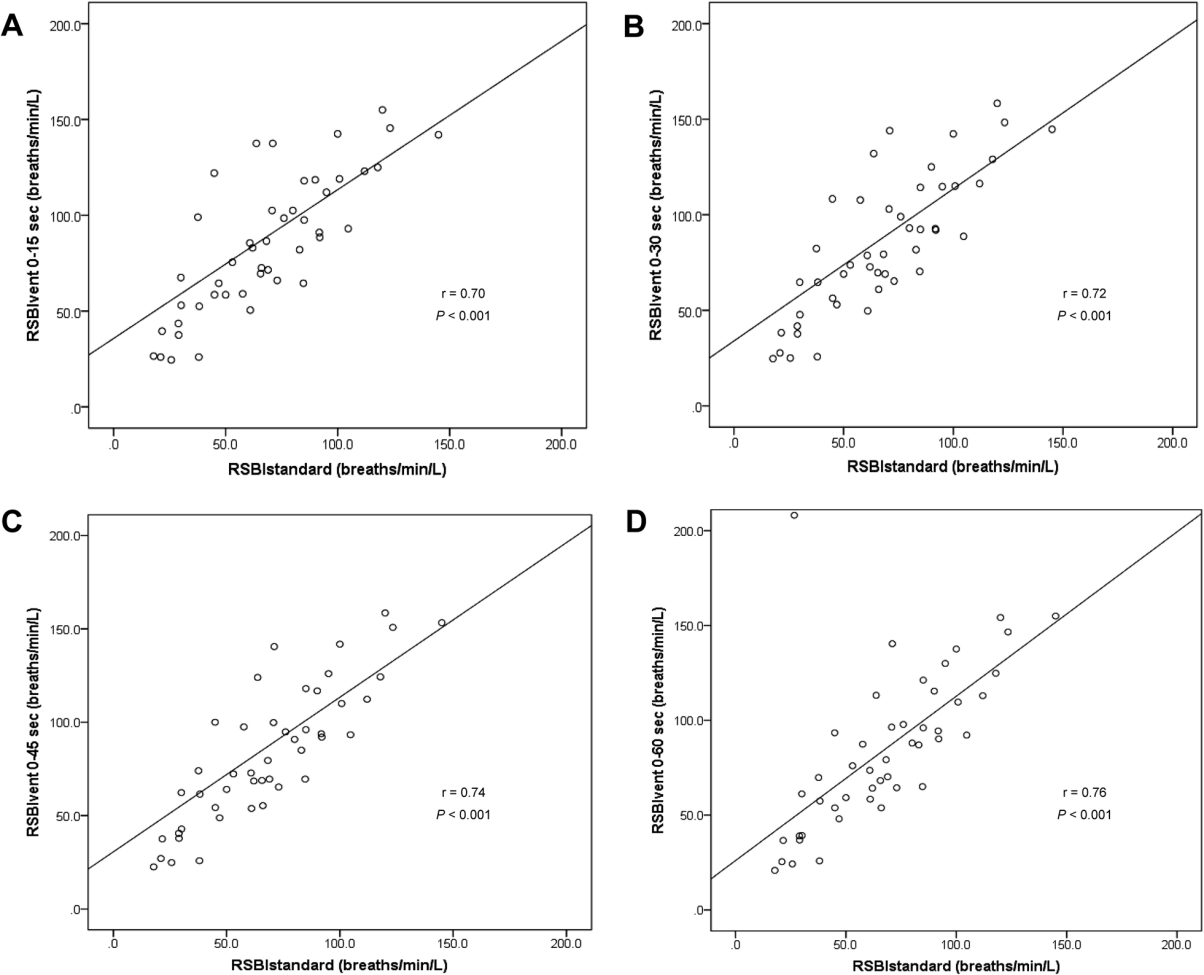
Correlation between rapid shallow breathing index (RSBI) by Wright spirometer (RSBI_standard_) and the average RSBI value displayed by the ventilator (RSBI_vent_): **a** At 0–15 s, **b** At 0–30 s, **c** At 0–45 s, and **d** At 0–60 s

Correlation analysis using Spearman’s correlation coefficient revealed a strong statistically significant correlation between the average value of RSBI_vent_ at different time points and the RSBI_standard_ value (Table 3). The average value of RSBI_vent_ from 0 to 60 s demonstrated the best correlation with RSBI_standard_ (*r* = 0.76; *p* < 0.001) (Fig. 2).

**Table 3:** Spearman’s Correlation coefficient and intraclass correlation coefficient between the rapid shallow breathing index measured by the Wright spirometer and the average rapid shallow breathing index value displayed by the ventilator at different time points

	*r*	*P* value	ICC	95% CI	*P* value
Average RSBI_vent_ at different time points					
0–15 s	0.70	< 0.001	0.73	0.52–0.85	< 0.001
0–30 s	0.72	< 0.001	0.74	0.53–0.85	< 0.001
0–45 s	0.74	< 0.001	0.73	0.52–0.85	< 0.001
0–60 s	0.76	< 0.001	0.79	0.61–0.88	< 0.001

*CI* confidence interval, *ICC* intraclass correlation coefficient, *r* Spearman’s correlation coefficient, *RSBI*_*standard*_ rapid shallow breathing index measured by the Wright spirometer, *RSBI*_*vent*_ rapid shallow breathing index displayed by the ventilator

Intraclass correlation coefficient demonstrated moderate to strong significant agreement between the average value of RSBI_vent_ at different time points and RSBI_standard_ (Table 3), and the average value of RSBI_vent_ from 0–60 s had highest agreement with RSBI_standard_ (ICC = 0.79, 95% CI 0.61–0.88; *p* < 0.001).

**Fig. 3:**
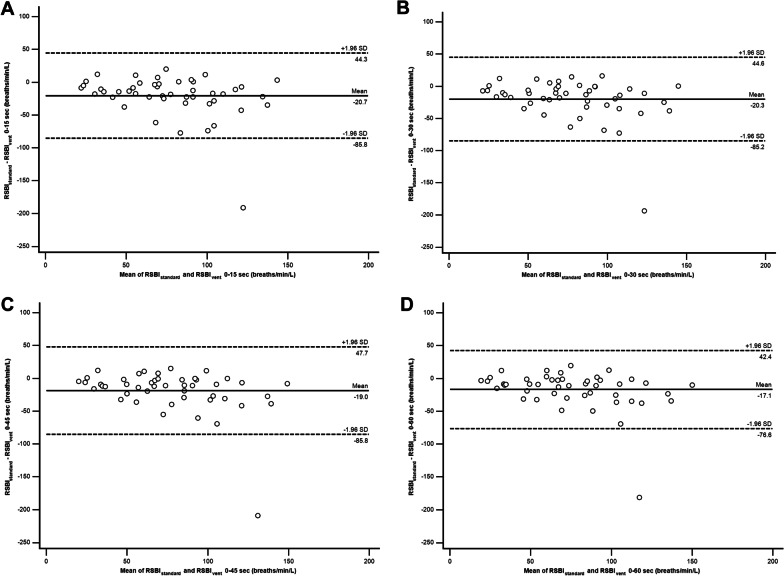
Bland–Altman plot analysis of agreement between rapid shallow breathing index (RSBI) by Wright spirometer (RSBI_standard_) and the average value of RSBI displayed by the ventilator (RSBI_vent_): **a** At 0–15 s, **b** At 0–30 s, **c** At 0–45 s, and **d** At 0–60 s

The mean difference between RSBI_standard_ and the average value of RSBI_vent_ at different time points is shown in Table 4. The average value of RSBI_vent_ from 0–60 s demonstrated the best agreement with RSBI_standard_ (mean difference between RSBI_standard_ and RSBI_vent_ from 0 to 60 s = −17.1 breaths/min/L, with a lower limit of − 76.6 breaths/min/L, and an upper limit of 42.4 breaths/min/L) (Fig. 3).

**Table 4:** Agreement of rapid shallow breathing index value measured by Wright spirometer and the average rapid shallow breathing index value displayed by the ventilator at different time points

	Mean difference	Upper limit	Lower limit
RSBI_standard_—RSBI_vent_ 0–15 s	− 20.7	44.3	− 85.8
RSBI_standard_—RSBI_vent_ 0–30 s	− 20.3	44.6	− 85.2
RSBI_standard_—RSBI_vent_ 0–45 s	− 19.0	47.7	− 85.8
RSBI_standard_—RSBI_vent_ 0–60 s	− 17.1	42.4	− 76.6

*RSBI*_*standard*_ rapid shallow breathing index measured by the Wright spirometer, *RSBI*_*vent*_ rapid shallow breathing index displayed by the ventilator

## Discussion

The results of this study showed that the ventilator significantly overestimated the RSBI value compared to the standard technique of RSBI measurement by Wright spirometer. However, using the average value of RSBI_vent_ from multiple different time points improved the accuracy of the RSBI_vent_ measurement. The average RSBI_vent_ value among 5 RSBI values (0, 0–15, 0–30, 0–45, and 0–60 s) measured during 0–60 s demonstrated the best correlation and agreement with RSBI_standard_.

Weaning from mechanical ventilation is an important process in critically ill patients after they recover from acute respiratory failure. Assessment of readiness to wean is the first step, and several parameters have been used to assess pulmonary function and to predict weaning outcome, such as breathing frequency, minute ventilation, maximum inspiratory pressure, and RSBI [13, 15]. RSBI, which is the ratio of breathing frequency divided by average tidal volume in one minute, is the most commonly used parameter in routine clinical practice, and a cut-off value below 105 breaths/min/L can predict the likelihood of weaning success [12, 16, 17].

Measurement of RSBI by Wright spirometer is a gold standard technique; however, it requires a special instrument that might not be available at the bedside. In addition, disconnecting the patient from the ventilator for a few minutes is required, and this may be harmful due to loss of PEEP effect and risk of contamination of the breathing tube and circuit. Measurement of RSBI using the value displayed by the ventilator during unassisted breathing such as flow-by method may be used to avoid the limitations of measuring RSBI by Wright spirometer. Furthermore, recent weaning guideline recommends using low pressure augmentation instead of T-piece or continuous positive airway pressure mode during SBT [18] then continuing pressure delivery with low pressure augmentation SBT after evaluating RSBI using the ventilator display would be preferred because disconnecting the patient from the ventilator is unnecessary.

Many modern ICU ventilators can measure and display the RSBI value; however, the displayed value may vary breath by breath according to both the algorithm used to calculate the RSBI value and patient’s breathing pattern. Other factors, such as pressure augmentation, PEEP, and a bias flow may also affect the measurement of RSBI. Several studies reported the RSBI value to be significantly lower when applying low level of pressure support and/or PEEP compared to unassisted breathing [19–23]. A bias flow in the flow trigger system may also influence the measurement of RSBI. A study by Kheir, et al.[24] found the RSBI value measured by the ventilator in the flow trigger mode to be significantly lower than during unassisted breathing. This can be explained by the effect of a bias flow in the flow trigger system that provides a small amount of pressure support that decreases work of breathing [25].

Very few studies have evaluated the accuracy of RSBI measured by the ventilator compared to the standard technique using Wright spirometer under the same ventilator condition at the same time. Patel et al. [23] measured RSBI during a flow-by mode using two different techniques, including the values measured and displayed by the ventilator, and the value measured by Wright spirometer attached to the expiratory port of the ventilator, in 91 subjects. They found no significant difference in RSBI between the two techniques; however, the measurements were not performed simultaneously. Lessa et al. [26] evaluated agreement between RSBI displayed by the ventilator during low level pressure support and digital ventilometer when the patient was disconnected from the ventilator in 22 subjects after postcardiac surgery. They found the RSBI to be significantly different between the two methods, but high agreement for RSBI, breathing frequency, and minute ventilation were still observed. In contrast, a study by de Sousa et al. [27] compared RSBI calculated by the ventilator during pressure support and PEEP of 5 cmH_2_O and traditional method by spirometer. They found a strong correlation and intraobserver variation coefficient between the two methods.

Despite our finding that the RSBI value measured and displayed by the ventilator was consistently significantly higher than the RSBI value measured by the standard technique by Wright spirometer, we also found that adding more values of RSBI at different time points improved the accuracy of RSBI displayed by the ventilator compared to the standard technique. The measurement of RSBI from the two techniques in our study was performed simultaneously with same ventilator settings by the same examiner. These factors should improve the reliability and accuracy of our results compared to previous studies that made non-simultaneous comparisons and used different ventilator settings. Using the RSBI value measured and displayed by the ventilator is more convenient than the standard technique because it does not require a special instrument, and it can be performed without disconnecting the patient from the ventilator; however, using only a single ventilator-generated RSBI value may lead to an insufficiently accurate prediction of weaning outcome. We found that using the average of 5 ventilator-generated RSBI values during 0–60 s (0, 0–15, 0–30, 0–45, and 0–60 s) improves the correlation between RSBI_vent_ and gold standard RSBI_standard_. In addition, continuous monitoring (longer than 1 min) of RSBI using the value displayed by the ventilator may be more appropriate than using Wright spirometer in patient who is at risk for SBT failure (i.e. RSBI value between 90 and 110 breaths/min/L) because disconnecting the patient from the ventilator is needed.

### Strength and limitations

The strength of this study is that this is the first study to compare these two RSBI measurement techniques simultaneously in patients deemed ready to wean from ventilatory support. However, our study has some mentionable limitations. First, this is a single center study. Second, only one brand of ventilator was used to evaluate the accuracy and reliability of RSBI_vent_ compared to the standard technique, so our findings may not be generalizable to other ventilator brands. Third, all measurements were done by the same examiner then the lack of interobserver comparison would limit the translatability into clinical practice. Last, we used a flow-by method during the measurement of RSBI. Using this method, the ventilator may deliver a small amount of pressure support, which means that the RSBI value might be lower than unassisted breathing.

## Conclusions

The results of this study revealed that the ventilator significantly overestimates the RSBI value compared to the standard technique by Wright spirometer. The average RSBI_vent_ value among 5 time points (0, 15, 30, 45, and 60 s) was found to have the best correlation with RSBI_standard_.

## Data Availability

The datasets used and analyzed for this study are available from the corresponding author on reasonable request.

## Abbreviations

APACHE: Acute Physiologic and Chronic Health Evaluation
CI: Confidence interval
FiO_2_: Inspired oxygen fraction
ICC: Intraclass correlation coefficient
ICU: Intensive care unit
PaO_2_: Arterial partial pressure of oxygen
PEEP: Positive end-expiratory pressure
*r*: Spearman’s correlation coefficient
RSBI: Rapid shallow breathing index
RSBI_standard_: RSBI measured by Wright spirometer
RSBI_vent_: RSBI displayed by the ventilator
SBT: Spontaneous breathing trial;
SpO_2_: Oxygen saturation by pulse oximetry
